# The Effectiveness of Short-Term Group Psychoeducation for Patients With Bipolar Disorder in Japan

**DOI:** 10.7759/cureus.76441

**Published:** 2024-12-26

**Authors:** Atsuko Inoue, Sayaka Kobayashi, Hidehiro Oshibuchi, Kaoru Tsuji, Ken Inada, Katsuji Nishimura

**Affiliations:** 1 Department of Psychiatry, Tokyo Women's Medical University, Tokyo, JPN; 2 Department of Psychiatry, Saitama Medical Center, Saitama Medical University, Tokyo, JPN; 3 Department of Psychiatry, Kitasato University School of Medicine, Sagamihara, JPN

**Keywords:** bipolar disorder, group psychoeducation, relapse prevention, self-efficacy, short version

## Abstract

Introduction

Psychoeducation is a form of psychosocial treatment with proven efficacy in preventing the relapse of bipolar disorder (BD). However, the effectiveness of psychoeducation has not been verified in Japan. We aimed to examine the effect of a brief group psychoeducation course (eight-session long) on relapse prevention in Japanese patients with BD and associated factors.

Methods

This single-arm, mirror-image study included 55 patients with BD. To verify the effectiveness of relapse prevention, the numbers of hospitalizations during the two years before and two years after participation were compared. Manic and depressive symptoms, treatment satisfaction, and self-efficacy (SE) in coping with illness were also assessed.

Results

The number of hospitalizations for the 47 participants who completed the program decreased significantly after psychoeducation. Overall, SE improved significantly after participation, but not among the participants who were hospitalized during the observation period. The hospitalized participants were significantly older, had more hospitalizations during the two years before participation, and had lower SE after participation than the non-hospitalized participants. They also exhibited higher depression levels after participation than before participation.

Limitations

The results of this single-arm, single-center study should be confirmed in future randomized controlled trials or other studies with a larger number of patients to demonstrate their generalizability.

Conclusions

Brief group psychoeducation courses may decrease relapse among Japanese patients with BD. A high number of hospitalizations prior to the psychoeducation program, older age, more severe depressive symptoms after participation than before participation, and low SE after participation could be associated with relapse.

## Introduction

Bipolar disorder (BD) is a chronic, life-altering psychiatric disorder. A US study reported a lifetime combined prevalence rate of 3.9% for type I and type II disorders [1]. In Japan, an epidemiological study reported a non-negligible estimated overall lifetime prevalence of 0.6% [2] for bipolar disorder. Advances in treatment, including the development of mood stabilizers, have led to favorable prognoses and social remission in bipolar disorder. However, repeated recurrence is a major problem in patients with bipolar disorder. Refractory cases significantly reduce health-related quality of life, decrease labor productivity, and increase associated costs [2]. Suicide rates in patients with bipolar disorder are also higher than those in patients with unipolar depression [3]. To prevent recurrence, patients need to improve their disease management strategies and reduce treatment non-adherence. Psychosocial therapy is important for this purpose, and in recent years, a combined approach based on pharmacotherapy and psychotherapy has been used [4,5].

Psychoeducation is a psychosocial therapy that helps individuals with unacceptable illnesses, such as mental illnesses. This supports independent living by providing correct knowledge and information while taking psychological aspects into consideration and helping people develop skills to cope with various problems caused by illness and disability [6]. The target population has expanded to include psychiatric disorders such as schizophrenia and depression [7] and physical disorders such as cancer [8], and bipolar disorder is one of these disorders.

Currently, psychoeducation for bipolar disorder is known as one of the psychosocial therapies that have been proven to be effective. Several randomized controlled trials and systematic reviews have shown that group psychoeducation for bipolar disorder influences relapse prevention [9,10]. In recent years, short-term psychoeducational interventions have also been proposed. For example, group psychoeducation with as few as four sessions has been shown to be effective in reducing the number of hospitalizations and emergency visits among patients with bipolar I disorder [11]. A systematic review and network meta-analysis revealed that compared with similar strategies in an individual format, psychoeducation in a family or group format was associated with reduced recurrence. Furthermore, compared with standard-length psychoeducation programs, brief psychoeducation courses were associated with higher participant retention rates [5]. These results indicate that group psychoeducation conducted over a short period of time has a relapse-prevention effect while being cost-effective. However, to the best of our knowledge, the effectiveness of group psychoeducation for bipolar disorder has not been verified in Japan.

Recently, some studies examined how patients with bipolar disorders benefit from psychoeducation. One study reported that patients with seven or more episodes prior to participating in psychoeducation did not show any significant improvement in the time to relapse [12]. Another study that examined response and non-response to group psychoeducation found that younger age at diagnosis of bipolar disorder, low cyclothymic temperament score, and male gender were significantly associated with increased responsiveness to psychoeducation [13]. These results reaffirm the importance of early detection and treatment of bipolar disorder. However, to our knowledge, no variables related to relapse prevention that could be influenced by the addition of psychoeducation to the usual treatments have yet been reported.

In this study, we aimed to confirm the effectiveness of short-term group psychoeducation on relapse prevention in patients with bipolar disorder in Japan. In addition, by comparing the relapse and non-relapse groups, we identified potential factors that could lead to relapse prevention.

## Materials and methods

Study design and procedure

This was a single-arm, mirror-image study with a two-year follow-up. As a preliminary step to a comparative study with a usual care group, a single-arm study was conducted to examine the efficacy and safety of the group intervention and the acceptability of the program to the participants at an early stage. Participants were enrolled in a group psychoeducation program in addition to their standard treatment. The primary outcome was the number of hospitalizations, which was recorded to determine the recurrence rate during the two years before and the two years after the completion of the psychoeducation program. Secondary outcomes were also evaluated.

The participants underwent an initial evaluation one week before the start of the psychoeducation course and an exit evaluation after the final session. Thereafter, they underwent follow-up evaluations every two months for two years. Follow-up was conducted primarily by the attending psychiatrist during regular office visits. If the participant was unable to come to the clinic on the scheduled day, the evaluation was conducted by a psychologist at a later date.

During participation in the study, the patients received their usual treatment from their primary care physician. In the event of symptom worsening, the physician decided whether the participant could continue participation in the program. Even in case of discontinuation, participants could return to the program or take supplementary classes after their condition had stabilized, provided their attending physician gave them permission to do so.

Ethical Considerations

This study was approved by the Research Ethics Committee of Tokyo Women's Medical University (approval number: 141003) on December 22, 2014, and was performed in accordance with the Declaration of Helsinki. All participants were fully informed and agreed to participate in the study. The public trial pre-registration was not required by the Research Ethics Committee. This study was registered in the University Hospital Medical Information Network (UMIN000028052, 2017/07/24).

The study adheres to the Consolidated Standards of Reporting Trials (CONSORT) guidelines for reporting clinical trials.

Participants

Patients diagnosed with bipolar disorder according to the Diagnostic and Statistical Manual of Mental Disorders (DSM)-IV-TR or DSM-5 [14,15] and attending Tokyo Women's Medical University Hospital were eligible for recruitment.

The inclusion criteria were age ≥ 20 years, receiving treatment for at least two years, not experiencing manic/depressive episodes at the time of recruitment, and being able to provide written informed consent to participate in the study on a voluntary basis.

Patients were excluded from the study if they were judged to be at risk of harm to themselves or others at the time of participation, had intellectual disabilities, had serious physical complications, or had impaired capacity to consent as judged by a psychiatrist, as well as if they were deemed unsuitable for inclusion by the principal investigator, in which the patient's medical condition was precarious owing to a comorbid diagnosis other than bipolar disorder or in which the patient had difficulty attending sessions regularly for work or residential circumstances.

Recruitment was conducted between January 2015 and July 2019. Permission from attending psychiatrists was required for participation in the study. Based on the inclusion and exclusion criteria described above, patients eligible for the program were referred to the program by their attending psychiatrists and encouraged to participate. Simultaneously, posters outlining the psychoeducation program were displayed in the outpatient waiting room so patients who wished to participate could do so after consultation with their attending psychiatrists.

Intervention psychoeducation program

We developed a shortened psychoeducation program based on Colom and Vieta's 21-session psychoeducation program [16]. The original program consisted of 21 sessions, but this program was designed to have eight sessions of 100 minutes each so that the program could be conducted on an outpatient schedule. Generally, the sessions were held once every two weeks and completed in four months. The psychoeducation courses were provided in a closed group format and led by two psychologists with more than 10 years of experience in psychoeducation and group psychotherapy and one or two psychiatrists acting as facilitators. Each session consisted of informational sessions using a textbook and group discussion sessions.

An overview of the program is presented in Table 1. In addition to the content covered in the original version, the themes of "how to deal with hospitals and staff" and "how to deal with people close to you" were added, as requested by the participants in the pilot study.

**Table 1:** Course content of the short-term group psychoeducation program for bipolar disorder

Session	Title	Contents
1	What is bipolar disorder?	Causes and triggers, symptoms of mania and depression, course, life chart
2	What to know about medication	Types and effects of medications used, side effects and how to deal with them, pregnancy/childbirth, and medications
3	What you need to know about living with the disease	How to deal with medical institutions, the relationships with people close to you, and hereditary issues
4	Alternative therapies and psychoactive substances	Alternative therapies, caffeine, alcohol, etc.
5	Early detection and response to episodes (mania)	Early symptoms and prodromal signs and what to do when you notice them
6	Early detection and response to episodes (depression) and the importance of a regular lifestyle	Early symptoms and prodromal signs, what to do when you notice them, the relationship between sleep and symptoms, and the rhythm of life
7	Stress control	Stress and coping
8	Problem-solving strategies	Practice problem-solving methods (group work) and concluding summary

The patients were examined individually by a psychiatrist before the start of each session, and only after it was confirmed that their condition had not worsened, they were allowed to participate in the session. If a participant missed a session, an individual catch-up session was provided.

Assessments

Primary Outcome Measure

The number of psychiatric hospitalizations due to bipolar disorder was evaluated as a measure of relapse prevention effectiveness. The state of the patient at the time of admission was recorded without distinguishing between manic and depressive episodes. The number of hospitalizations during the two years prior to psychoeducation was retrospectively extracted from the medical history and compared with the number of new hospitalizations during the two years after psychoeducation. For those who were hospitalized at the time of participation, the hospitalizations were counted as pre-participation hospitalizations, and further hospitalizations following discharge were counted as post-participation hospitalizations.

Secondary Outcome Measures

Percentage of hospitalizations: The percentage of people who experienced hospitalization within two years prior to and after psychoeducation participation was calculated and compared. Of those who had been hospitalized during the two years prior to participation, we calculated the percentage of people who were also hospitalized during the two years after participation.

Symptom assessments: Manic symptoms were measured using the Japanese version [17] of the Young Mania Rating Scale (YMRS) [18], an 11-item symptom rating interview. Depressive symptoms were measured using the Japanese version [19] of the 17-item Hamilton Depression Rating Scale (HAM-D17) [20], a structured interview measuring depression severity. These assessments were carried out by the attending psychiatrist or psychologist at regular office visits before and at the end of the psychoeducation program and during the follow-up period.

Patient satisfaction with the program: At the end of the psychoeducation program, participants' satisfaction was evaluated using the Japanese version of the Client Satisfaction Questionnaire (CSQ-8J) [21]. Originally, a score of 25 or higher denoted relatively high satisfaction [21]. In this study, a score of 25 or higher denoted high satisfaction with the program.

Self-efficacy (SE) of living with bipolar disorder: Self-efficacy is an individual's conviction that one can successfully execute the behavior required to produce the outcomes [22]. An original five-item self-efficacy scale was developed and used to measure the patients' confidence in coping with bipolar disorder. To maintain the validity of the content, the items were drafted by two clinical psychologists who were familiar with the content and goals of the program. The following items were used: "I know a lot about my illness (symptoms and characteristics)," "I know a lot about the treatment and coping methods of my illness," "I am coping well with my illness now," "I think I can manage to cope with my illness in the future," and "I am confident that I can control my illness by myself." Higher scores indicate higher self-efficacy. The total score of the five items was calculated. The participants were asked to respond before the start of the program, at the end of the program, and two years after the end of the program.

Social life: Data on the participants' employment status, marital status, and whether they were living with cohabitants at the time of their participation in the psychoeducation program were extracted from their medical records.

Changes in medication: Medical records were examined to assess changes in doses of mood stabilizer and chlorpromazine-equivalent antipsychotic [23] use among patients before participation in the psychoeducation program and during the follow-up period. It was also determined if the patients discontinued medication without consultation.

Statistical analysis

The mean numbers of hospitalizations during the two years before and after the psychoeducation program were examined using the t-test, and Cohen's d was calculated to determine the effect size. The χ-squared test was used to compare the proportions of hospitalizations during the two years before and after the psychoeducation program.

One-way analysis of variance with repeated measures was used to examine whether there were differences in the YMRS, HAM-D, and self-efficacy scores before, after, and two years after the completion of the psychoeducation program. Multiple comparisons were made by using Dunnett's method when significant differences were observed.

To determine satisfaction with the program, the means and standard deviations (SDs) of the CSQ-8J scores at the end of the psychoeducation program were calculated. To determine factors associated with relapse, the data on hospitalization (yes or no) and participant demographics (gender, disease type, marital status, cohabitation status, and employment status) for two years following psychoeducation were analyzed with a χ-squared test. A t-test was also conducted with hospitalization as the independent variable and symptom rating scale and self-efficacy scale scores before and after participation, satisfaction, and the number of missed sessions as dependent variables. Cohen's d was calculated to determine these effect sizes. When the normality of distribution could not be confirmed, Mann-Whitney's U test was performed. Similar analyses were conducted on the dose of mood stabilizers and chlorpromazine-equivalent antipsychotics before and two years after participation in the psychoeducation program. In addition, YMRS, HAM-D, and self-effectiveness scores before and after participation were compared between the groups with and without hospitalization using the corresponding t-tests. When the normality of distribution could not be confirmed, Wilcoxon signed-rank tests were performed. Similarly, the dose of mood stabilizers and chlorpromazine-equivalent antipsychotics before and two years after participation were compared using a paired t-test. SPSS software version 28.0.1.0 (142) (IBM Corp., Armonk, NY) was used for all statistical analyses. A two-sided p < 0.05 was considered statistically significant.

## Results

Participant characteristics

**Figure 1:**
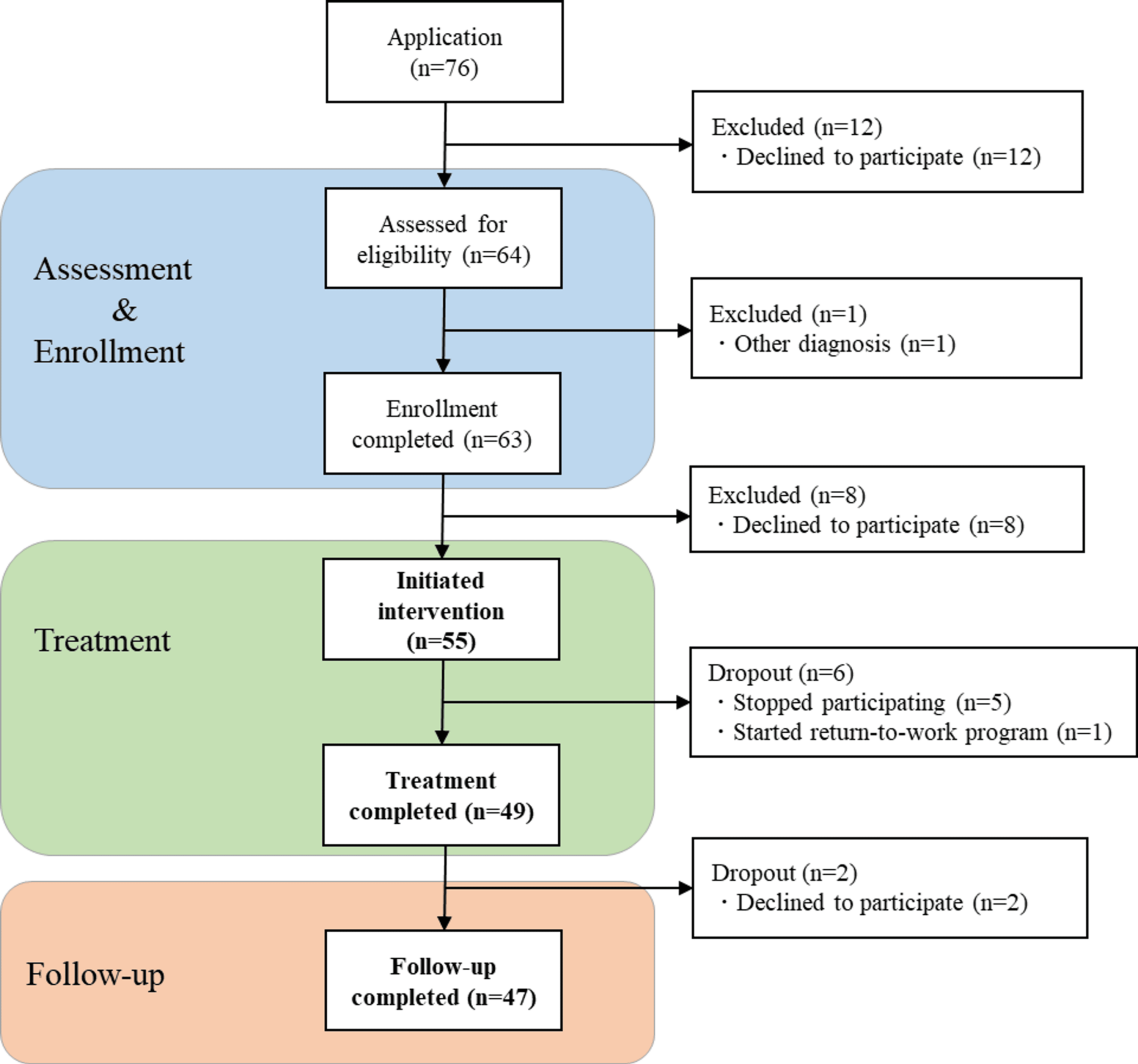
Study flowchart of participants from application to follow-up completion

A flowchart of participant inclusion is shown in Figure 1. A total of 76 people applied for the program, and 55 who met the criteria participated. Those who withdrew their participation before the start of the psychoeducation program were excluded. During the program, five participants dropped out (deterioration of their condition, four participants; unknown reasons, one participant). Another participant withdrew from the psychoeducation program because he was scheduled to participate in a return-to-work program. Finally, 49 participants completed the program (completion rate: 89.1%).

Two participants withdrew from the study during the follow-up period; hence, 47 (85.4%) participants (17 men and 30 women) completed the program and follow-up and were included in the analysis. All participants were Japanese. Fifteen participants were diagnosed with bipolar I disorder and 32 participants with bipolar II disorder. Two (4.3%) participants were hospitalized at the time of participation. Table 2 shows the characteristics of the participants.

**Table 2:** Baseline characteristics of the participants *Number of people taking the drug, and mean and standard deviation of the dose they are taking in mg. SD: standard deviation

Variable	N = 47
Age, years (mean (SD) (range))	48.9 (11.8) (29-70)
Female (number (%))	30 (63.8)
Ethnicity, Japanese (number (%))	47 (100)
Diagnosis (number (%))	
Bipolar Ⅰ disorder	15 (31.9)
Bipolar Ⅱ disorder	32 (68.1)
Disease duration, years (mean (SD) (range))	16.5 (9.3) (3-40)
Medication* (multiple answers allowed)	
Mood stabilizers (number (%))	38 (80.9)
Lamotrigine (number, mean (SD))	18, 207.78 (126.78)
Lithium carbonate (number, mean (SD))	18, 661.11 (252.37)
Sodium valproate (number, mean (SD))	12, 853.33 (336.22)
Carbamazepine (number, mean (SD))	3, 733.33 (416.33)
Antipsychotics (number (%))	27 (57.4)
Chlorpromazine equivalent amount (mean (SD))	296.77 (229.26)
Antidepressants (number (%))	4 (8.5%)
No use of the three medication types (number (%))	3 (6.4%)
Comorbidities affecting mental health (number (%))	
Alcohol use disorder	3 (6.4%)
Sedative, hypnotic, or anxiolytic use disorder	1 (2.1%)
Personality disorder	1 (2.1%)
Social anxiety disorder	2 (4.3%)
Panic disorder	1 (2.1%)
Specific phobia	1 (2.1%)
Obsessive-compulsive disorder	1 (2.1%)
Posttraumatic stress disorder	1 (2.1%)
Attention-deficit/hyperactivity disorder	1 (2.1%)
Premenstrual dysphoric disorder	1 (2.1%)
Epilepsy	1 (2.1%)
Paroxysmal kinesigenic choreoathetosis	1 (2.1%)
Marital status (number (%))	
Single	13 (27.7)
Married	27 (57.4)
Divorced	6 (12.8)
Bereaved	1 (2.1)
Living with someone (number (%))	
Yes	32 (82.1)
No	7 (17.9)
Employment status (number (%))	
Employed (full time)	8 (17.0)
Employed (part time)	3 (6.4)
Housework	15 (31.9)
Unemployed	18 (38.3)
Others	3 (6.4)

Primary outcome measure

**Figure 2:**
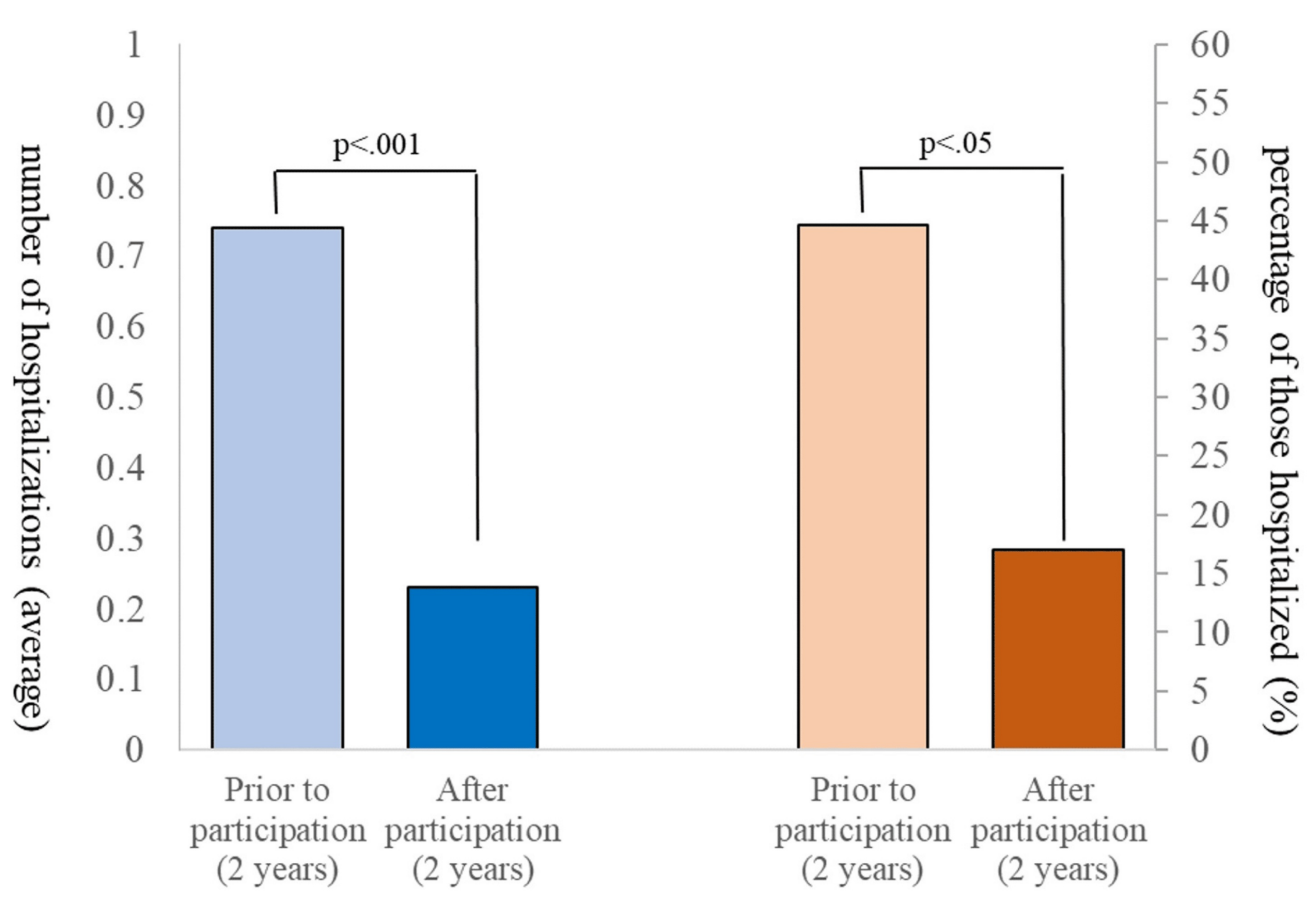
Changes in the number of hospitalizations and hospitalization rates The average number of hospitalizations and the percentage of participants who were hospitalized during the two years before and after participation in the psychoeducation program were compared.

The mean number of hospitalizations per person before and after participation in the psychoeducation program and the percentage of those who were hospitalized are shown in Figure 2. The mean number of hospitalizations per person was 0.74 ± 1.24 (SD) during the two years before participation and 0.23 ± 0.60 (SD) during the two years after participation, showing a significant decrease (t(46) = 3.02, p = 0.001, Cohen's d = 0.441, 95% confidence interval = 0.139-0.738).

When analyzing the data of only those who were hospitalized during the two years prior to participation (n = 21), we observed a significant decrease in the average number of hospitalizations between the two-year period prior to participation (1.67 ± 1.39 (SD)) and the two-year period after program completion (0.48 ± 0.81 (SD); t(20) = 3.71, p = 0.001, Cohen's d = 0.81, 95% confidence interval = 0.307-1.297).

Secondary outcome measures

Twenty-one (44.68%) patients were hospitalized during the two years prior to participation, and eight (17.02%) were hospitalized during the two years after the completion of the program, showing a significant difference (p = 0.015). Of the 21 participants who had been hospitalized during the two years prior to participation, seven were also hospitalized during the two years after participation (33.33%). One person who was hospitalized during participation was also hospitalized after participation. Table 3 shows the characteristics of those who were hospitalized during the two years after the end of the program.

**Table 3:** Characteristics of the hospitalized participants *Data from seven participants who were hospitalized during the two years prior to participation and also during the two years after participation. SD: standard deviation

Variable	N = 8
Last hospitalization before intervention, days (mean (SD))	47.63 (27.39)
First hospitalization after intervention, days (mean (SD))	31.25 (20.11)
Time to first hospitalization after intervention, days (mean (SD) (range))	158.25 (158.00) (-14-439)
Number of hospitalizations after intervention (mean (SD) (range))	1.38 (0.74) (1-3)
Reasons for first hospitalizations after intervention	
Manic (number (%))	3 (37.5)
Depressive (number (%))	5 (62.5)

The results of symptom ratings, self-efficacy, and treatment satisfaction are shown in Table 4. There were no significant differences in the YMRS and HAM-D scores obtained before, after, and two years after the completion of the psychoeducation program. On the contrary, there were significant differences in the self-efficacy scores obtained before, after, and two years after the completion of the program, where multiple comparisons revealed a significant difference of p < 0.001 between pre-participation scores and all post-participation scores. The mean CSQ-8J score for satisfaction at the end of psychoeducation was 27.51 ± 2.71.

**Table 4:** Score differences by time before, after, and two years after the completion of the psychoeducation program (N = 47) ***p < 0.001 #: pre < post = after two years CSQ-J: Client Satisfaction Questionnaire Japanese version, HAM-D17: 17-item Hamilton Depression Rating Scale, YMRS: Young Mania Rating Scale, SD: standard deviation

	Pre (mean (SD))	Post (mean (SD))	After 2 years (mean (SD))	F	p-value (adjusted)
HAM-D17	4.74 (3.77)	4.91 (3.71)	4.49 (4.93)	0.16	0.852
YMRS	2.89 (3.40)	2.51 (2.97)	2.21 (3.03)	0.714	0.492
Self-efficacy scale	12.09 (2.69)	13.89 (2.50)	14.38 (2.51)	21.882	<0.00***#
CSQ-J	-	27.51 (2.71)	-	-	-

Two years after the psychoeducation program, none of the patients had self-interrupted their medication. Four patients had stable conditions and discontinued their medication during the observation period with the approval of their attending psychiatrists because of planned pregnancy. The analysis of doses was performed for 42 patients, excluding five patients whose medication doses were unknown due to transfer to different hospitals. There were no significant differences in the doses of mood stabilizers and chlorpromazine-equivalent antipsychotics taken before and two years after the psychoeducation program.

Indicators of hospitalization two years after program completion

The demographic characteristics and secondary outcome measures according to hospitalization status two years after the completion of psychoeducation are shown in Table 5. There were no significant differences in gender, disease type, marital status, cohabitation, employment status, duration of illness, HAM-D scores after participation, YMRS scores before and after participation, and the number of missed sessions between the 39 participants who were not hospitalized and the eight participants who were hospitalized. However, the eight participants who were hospitalized were significantly older, had more hospitalizations during the two years prior to the psychoeducation program, and had lower self-efficacy scores immediately after psychoeducation participation than the 39 participants who were not hospitalized. In addition, there was a significant difference in HAM-D scores between the two groups prior to participation.

**Table 5:** Comparison between groups with and without hospitalization during the two years after psychoeducation *p < 0.05, **p < 0.01 Mann-Whitney's U test was used for the number of hospitalizations during the two years before psychoeducation, the number of missed sessions, and CSQ-J, HAMD17 (before and after participation), and YMRS (before and after participation) scores because the normality of the distributions could not be confirmed. For the χ-square test, the exact probability using Fisher's direct method was used due to the small sample size. CSQ-J: Client Satisfaction Questionnaire Japanese version, HAM-D17: 17-item Hamilton Depression Rating Scale, YMRS: Young Mania Rating Scale

Variable	Non-hospitalization (n = 39)	Hospitalization (n = 8)	χ^2^	t	U	p-value	Cohen's d
Attribution							
Female (number (%))	25 (64.1)	5 (62.5)	0.007			1.000	
Bipolar Ⅰ disorder (number (%))	11 (28.2)	4 (50.0)	1.451			0.245	
Married (number (%))	23 (59.0)	4 (50.0)	0.219			0.707	
Living without anyone (number (%))	7 (17.9)	3 (37.5)	1.515			0.340	
Unemployed (number (%))	14 (35.9)	4 (50.0)	0.559			0.692	
Age (years) (mean (SD))	47.26 (11.8)	56.75 (8.8)		-2.148		0.037*	-0.834
Disease duration, years (mean (SD))	15.44 (7.8)	21.50 (14.2)		-1.174		0.274	-0.668
Number of hospitalizations during the two years before psychoeducation (mean (SD))	0.56 (1.2)	1.63 (1.1)			254.000	0.004**	
Number of missed sessions (mean (SD))	0.62 (0.7)	0.50 (0.8)			141.500	0.687	
Measures (mean (SD))							
CSQ-8J	27.77 (2.7)	26.25 (2.5)			102.500	0.132	
HAM-D17 (pre)	5.28 (3.8)	2.13 (2.0)			72.500	0.016*	
HAM-D17 (post)	4.56 (3.6)	6.63 (4.0)			208.500	0.139	
YMRS (pre)	2.95 (3.6)	2.63 (2.4)			163.000	0.857	
YMRS (post)	2.33 (2.3)	3.38 (5.3)			157.500	0.967	
Self-efficacy scale (pre)	12.05 (2.8)	12.25 (2.4)		-0.188		0.852	-0.073
Self-efficacy scale (post)	14.27 (2.2)	12.00 (3.0)		2.465		0.018*	0.957

At the time of participation in the psychoeducation program, there was no significant difference in the doses of mood stabilizers and chlorpromazine-equivalent antipsychotics between the no-hospitalization group and the hospitalization group. However, two years after completing the program, the dose of chlorpromazine-equivalent antipsychotics was significantly higher in the hospitalized group (t (40) = 2.15, p = 0.038, d = 0.845), but not the dose of mood stabilizers.

Differences in the YMRS, HAM-D, and self-efficacy scores before and after psychoeducation were examined according to the presence or absence of hospitalization two years after participation. In the group without hospitalization, there were no significant differences in the distribution of the YMRS and HAM-D scores before and after participation, but self-efficacy scores were significantly higher (p < 0.001) after participation (median: 14 (interquartile range: 13-16)) than before participation (12 (9-14)). In contrast, in the inpatient group, there were no significant differences in the distribution of the YMRS and self-efficacy scores before and after participation in the psychoeducation program, but there were significant differences in the distribution of the HAM-D scores before (2 (0.25-3.0)) and after (5.5 (5.0-10.25)) participation (p = 0.042).

Thirty-four of 39 patients (excluding five patients whose dosage was unknown) who were not hospitalized during the two-year period showed no change in the dose of medications before and two years after the end of the psychoeducation program. For the inpatient group, only the chlorpromazine-equivalent antipsychotic dose showed a significant difference in their distribution in the Wilcoxon signed-rank test (median: 132.58 (interquartile range: 0.00-375.76) before participation, 351.52 (28.41-796.97) two years after participation, p = 0.042).

## Discussion

The short-term group psychoeducation program for patients with type I and type II bipolar disorder contributed to a significant decrease in the average number of hospitalizations from 0.74 to 0.23 and in the hospitalization rate from 44.68% to 17.02% during the two years after participation, compared with the two years before participation in the program. In addition, there was an improvement in self-efficacy in coping with illness after participation in the program.

The participants who were hospitalized during the two-year follow-up period were significantly older than those who were not hospitalized, had experienced more hospitalizations during the two years prior to participation, had higher HAM-D scores after participation than before participation, and had lower self-efficacy scores after participation. On the contrary, there were no significant differences with respect to gender, type I or type II bipolar disorder, duration of illness, YMRS scores before participation, employment/marriage or cohabitation status at the time of participation, the dose of medications, and program satisfaction. Those who were hospitalized during the follow-up period had significantly higher HAM-D scores after participation than before participation, as well as a significantly increased use of chlorpromazine-equivalent antipsychotics two years after completing psychoeducation. Those who were not hospitalized had significantly higher self-efficacy scores after participation than before participation.

Effectiveness of the program

The mean number of hospitalizations and the hospitalization rate significantly decreased during the two years after the psychoeducation program, suggesting that this program may have a relapse-preventive effect on patients with bipolar I and II disorders in Japan.

Few previous studies have examined the effect of short-term group psychoeducation on the number of hospitalizations; however, one study reported that for outpatients with bipolar I, the average number of hospitalizations during one year before and after four sessions of psychoeducation decreased from 0.7 to less than 0.2 [11]. The present study had a two-year follow-up period, and the hospitalization rate for patients with bipolar I disorder was higher than the 5.63% readmission rate observed by Chen et al. one year after eight sessions of psychoeducation [24]. However, their study included inpatients, and those results cannot be directly compared with our results because the criteria for hospitalization may differ between countries.

Patients with bipolar disorder have low self-efficacy in managing their illness and living environment [25]. The program in our study could increase participants' self-efficacy in living well with bipolar disorder. Self-efficacy is a factor that increases the likelihood of behavior change, and it is noteworthy that participation in psychoeducation affected this aspect.

Factors that can transform self-efficacy include accomplishments in performance, vicarious experience, verbal persuasion, and physiological states [22]. Listening to other participants' coping experiences, successful completion of the program with homework over a four-month period, and positive information and feedback may have contributed to the increased self-efficacy in participants. It has also been reported that increased self-efficacy following psychoeducation occurs provided there is extensive contact with peers and other people. Moreover, the participants themselves mentioned peer contact as a benefit [26]. Peer support has thus been shown to improve self-efficacy in patients with bipolar disorder [27]. Group psychoeducation offers an opportunity for peer support, where people with the same illness gather and face challenges together while listening to the experiences and innovative approaches of other participants. Contact with peers in our program may have also led to improved self-efficacy.

Program feasibility and usefulness

The completion rate for this program was 89.19%. As per previous studies, short-term group psychoeducation had a higher completion rate than longer-term psychoeducation, with a reported completion rate of 80.7% for all 21 outpatient sessions [28] and 91.7% for all eight sessions [29]. Our results are generally comparable to those of previous studies.

There was no worsening of the symptom rating scale scores before or after participation among the psychoeducation program completers, and only one (2.1%) person was hospitalized during program participation. We hypothesize that the patients were introduced to psychoeducation at a time when their symptoms were stable; hence, they could maintain the same stable state during their participation in the program and during the two years after participation. There was no association between the number of missed sessions and relapse. We believe that allowing participants to receive catch-up sessions functioned effectively. It is also noteworthy that the program satisfaction level of the participants was very high. These results suggest that our program is safe, effective, and convenient for use in clinical settings.

Factors associated with relapse

The group that was hospitalized during the follow-up period was older and had more hospitalizations during the two years prior to participation in the psychoeducation program than the non-hospitalized group. A higher number of episodes prior to participation has been associated with reduced responsiveness to psychoeducation [12]. The present results are consistent with those of previous studies, as hospitalization reflects the presence of severe episodes.

The HAM-D scores before participation were significantly lower in the inpatient group than in the non-inpatient group. Nevertheless, after participation, scores significantly increased in the inpatient group only. Hence, these large fluctuations in depression scores before and after participation in the program should be considered with caution.

Considering others' findings regarding the number of previous hospitalizations [12], it is possible that older patients who have been unstable for a long period of time before participating in the psychoeducation program and are particularly prone to fluctuating depressive symptoms may be less likely to benefit from psychoeducation. Therefore, it may be beneficial to provide psychoeducation as early as possible.

Furthermore, no improvement in self-efficacy after participation in the psychoeducation program was observed among those who were hospitalized during the follow-up period. Additional support may be needed for a group that, despite high program satisfaction, has not shown improvement in self-efficacy in dealing with their illness.

On the contrary, no significant differences were found in terms of gender, disease type, marital status, or cohabitation/employment status. Although previous studies have reported that men are more responsive to psychoeducation than women [13] and that patients with bipolar II are at higher risk of relapse than those with type I [30], no such trends were found in this study. To our knowledge, no prior studies have focused on the relationship between social status, such as cohabitation or employment, and response to psychoeducation. Further research on these related factors is needed.

Limitations

This single-arm study was conducted at a single institution with limited participants. As single-arm studies do not have a control group, it is not possible to demonstrate a causal relationship between the intervention and outcome. Moreover, the possibility that the applicability of the results is limited to a specific population or condition cannot be excluded. Therefore, confirmation through randomized controlled trials is necessary to demonstrate intervention effects and confirm the generalizability of the results. Moreover, further studies with more significant numbers of patients are needed to verify the factors contributing to the effectiveness of psychoeducation.

The results of this study were obtained only from patients who volunteered to participate, and the participants were highly motivated to learn. Furthermore, because only those who received permission from their attending psychiatrists were included in the study, their relationships with their attending psychiatrists possibly limited the number of participants. Therefore, the study did not fully demonstrate the effects of the program on bipolar disorder.

Reaching out to families and communities is an issue for the future.

## Conclusions

Eight sessions of brief group psychoeducation may reduce relapses among patients with type I and type II bipolar disorder in Japan. A higher number of hospitalizations before program participation, older age, and more severe depressive symptoms after participation were associated with relapse after participation. Improved self-efficacy through psychoeducation may help prevent relapse. Patients who report few depressive symptoms immediately before starting psychoeducation should be carefully monitored, including during their participation. When providing psychoeducation, it is necessary to recommend introducing it as early as possible after the onset of the disease and to consider more individualized support when the improvement of self-efficacy is insufficient.
